# Predictive Value of Ambulatory Objective Movement Measurement for Outcomes of Levodopa/Carbidopa Intestinal Gel Infusion

**DOI:** 10.3390/jpm12010027

**Published:** 2022-01-02

**Authors:** Gökçe Kilinçalp, Anne-Christine Sjöström, Barbro Eriksson, Björn Holmberg, Radu Constantinescu, Filip Bergquist

**Affiliations:** 1 Department of Neurology, Sahlgrenska University Hospital, 413 45 Göteborg, Sweden; gokce.kilincalp@vgregion.se (G.K.); anne-christine.sjostrom@vgregion.se (A.-C.S.); barbro.eriksson@neuro.gu.se (B.E.); bjorn.holmberg@vgregion.se (B.H.); radu.constantinescu@vgregion.se (R.C.); 2 Department of Pharmacology, Sahlgrenska Academy, University of Gothenburg, 405 30 Göteborg, Sweden

**Keywords:** ambulatory movement measurement, levodopa/carbidopa intestinal gel, Parkinson’s disease, motor fluctuations

## Abstract

Patients with Parkinson’s disease that may benefit from device-assisted therapy can be identified with guidelines like Navigate PD. The decision to offer advanced treatment and the choice of treatment modality are, however, not straightforward, and some patients respond less favorably to a chosen therapy. Measurements with the Parkinson Kinetigraph (PKG) can detect motor fluctuations and could therefore predict patients that respond better or worse to intestinal levodopa/carbidopa gel infusion (LCIG). In a retrospective analysis of 45 patients that had been selected to start LCIG between 2014 and 2020, the effects of baseline PKG and clinical characteristic on the outcome were determined with ordinal regression. Although all patients had been found to have handicapping medication-related symptom fluctuations, patients without clear objective off fluctuations in the baseline PKG had low odds ratio for success. Lower odds for success were also found with increasing age, whereas gender, medication intensity and baseline PKG summary scores (median bradykinesia and dyskinesia scores, fluctuation dyskinesia score and percent time with tremor) had no significant effect. Absence of easily identified off-periods in the PKG has a negative prognostic value for the effect of LCIG and could prompt noninvasive infusion evaluation before surgery.

## 1. Introduction

The most effective symptomatic treatment of Parkinson’s disease (PD) is levodopa. However, with disease progression levodopa treatment is associated with shortened effect duration (wearing off) and other symptom fluctuations related to variable levodopa plasma concentrations [1]. In selected persons with PD (pwPD), device-assisted treatments with continuous drug infusion or deep brain stimulation are effective measures to alleviate symptom fluctuations and improve quality of life [2,3,4,5]. The selection of patients who will clearly benefit is, however, not straightforward. Although there are consensus statements [6,7] on who should be considered for device-assisted therapy, the selection of pwPD that should eventually be offered device-assisted therapy and the choice of treatment modality may vary according to local practice. Although most pwPD started on device-assisted therapy have an excellent effect, a number of them will experience limited or no improvement in quality of life, even if the selection is made at specialized movement disorder centers [8,9,10].

Objective assessment of motor fluctuations with wearable sensors has recently been introduced for the clinical follow-up and evaluation of pwPD [11,12,13]. As is common with new techniques, the introduction has been driven by early adopters with a strong conviction regarding its usefulness, and there are few published studies of the effects of using such devices for managing pwPD [14,15,16,17]. A recent study demonstrated that information from one such device, the Parkinson Kinetigraph (PKG, Global Kinetics Corporation) could be used in a classifier algorithm to identify patients that should be considered for device-assisted therapy [17], thereby supplementing other previously proposed “rules of thumb” for identifying these patients, like 5–2–1 (at least five doses of levodopa, 2 h in the “off state” and/or 1 h of troublesome dyskinesia) [6,7]. We introduced PKG as a standard evaluation tool for PD in West Sweden when it was made available in 2013. Our own experience with using the PKG on unselected pwPD suggests that motor complications are very commonly detected, roughly in 50–60% of them [18].Despite the high prevalence of sensor detected fluctuations, not all pwPD that are referred for device-assisted therapy display such fluctuations. Some reasons can be that the patient experiences predominantly non-motor fluctuations or motor symptoms that are not detected with the wrist-worn PKG device, like freezing of the gait or axial dyskinesia or leg symptoms. Therefore, pwPD who report troubling symptom fluctuations are considered for device-assisted therapy even if fluctuations are not detected with the PKG device. In this retrospective study, we investigated if the results of the pre-assessment PKG or other clinical factors are associated with the outcome of levodopa/carbidopa intestinal gel infusion (LCIG) as evaluated with Clinical Global Impression improvement, CGI-I. We found lower odds for a good outcome in patients where off periods are not visually identified in the baseline PKG recordings and with increasing age.

## 2. Materials and Methods

All pwPD that had started treatment with LCIG at Sahlgrenska University Hospital since we started to use PKG in clinical practice in 2013 were identified from the local PKG database and medical records. Medical records from individuals who had performed a pretreatment PKG measurement within 12 months of start of LCIG were analyzed retrospectively. The inclusion criteria for the study were that a PKG measurement had been performed within 12 months before treatment start with LCIG and that a 12-month assessment of the effect was available in November 2021. Exclusion criteria were current or previous treatment with deep brain stimulation, severe dementia or comorbidity with a large detrimental effect on patient mobility or quality of life.

One of the authors (G.K.) analyzed the medical records available at the time of the pretreatment PKG, at treatment initiation, and in the time period 9 months to 15 months after infusion start. The overall outcome of LCIG was rated with Clinical Global Impression of Improvement (CGI-I) [19] based on information in the medical record closest to 12 months after treatment initiation. The improvement was assessed in relation to the baseline before treatment start. The seven grade CGI-I scale includes: (1) Very much improved, (2) Much improved, (3) Minimally improved, (4) No change, (5) Minimally worse, (6) Much worse and (7) Very much worse. Patients were then classified into the following three outcome groups: complete responders if CGI ≤ 2, partial responders if CGI = 3, and non-responders if CGI > 3.

**Figure 1:**
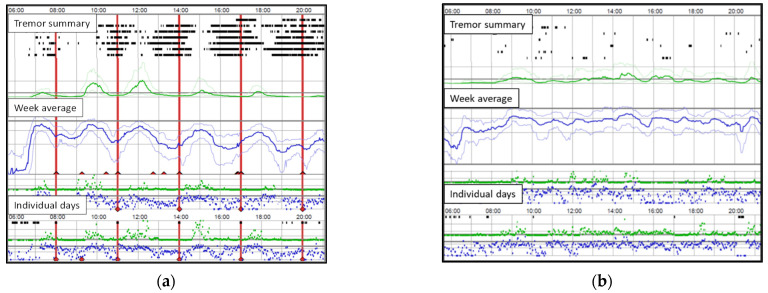
(**a**) PKG recordings with objective measure (OM) OFF periods reflected in the tremor pattern and medication locked BK wearing OFF in both averaged scores and on individual days. (**b**) PKG recording without OM OFF periods. Note that occasional inactivity periods can occur on individual days but do not qualify as OM OFF if there is no systematic relation to medication or if combined with sleep.

Clinical characteristics were derived from the medical records before and after 1 year of infusion. All dopaminergic medication was translated into levodopa equivalents (LED). PKG devices were worn for 6–10 days and mostly 24 h/day. In addition to standard summary measures from the PKG recordings (medians of bradykinesia BK and dyskinesia DK scores, fluctuation dyskinesia score and percent time with tremor during the daytime, 9–18), all recordings were visually assessed for signs of wearing off (bradykinesia worsening near medication times), which was annotated as Objective Measure (OM) OFF periods. PKG recordings were also used to estimate the average effect duration of individual levodopa doses, and the objective measure daily ON time (OM ON) was determined by visual assessment of the PKG. All visual evaluations of the PKG recordings were made by one of the authors (F.B.) who has >8 years of experience of clinical interpretation of PKG. Illustrations of PKG recordings with and without OM OFF are given in Figure 1. The corresponding information was collected from the latest available PKG from the first year after LCIG start in patients where that was available.

Data were analyzed with SPSS version 25 (IBM Corporation, Armonk, NY, USA). An ordinal regression model was used with the three outcome groups as the dependent variable; gender and presence/absence of OM OFF as independent factors and age; baseline PKG summary scores (median BK, DK, percent time with tremor and FDS); OM ON time; levodopa dose frequency and the natural logarithm of the total daily LED as covariates. The odds ratios for each factor and covariate were determined. Descriptive statistics of the data were given for each patient outcome category (complete responders, partial responders and non-responders). Nonparametric tests were used to evaluate changes in the outcome variables at one year (levodopa equivalent doses) and at the time of the follow-up PKG (change in the PKG summary scores, OM ON time and the absence of OM OFF periods). Values are given as the median (min–max) if not otherwise specified. Due to the retrospective and explorative nature of the study, no adjustment for multiple comparisons was made.

## 3. Results

### 3.1. Study Population

Data fulfilling inclusion/exclusion criteria was available in 45 patients: 16 females and 29 males (64.4% males). The mean (SD) interval between the pretreatment PKG and start of intestinal levodopa infusion was 8.0 (3.0) months. The baseline characteristics at the time of the pretreatment PKG are described in Table 1.

### 3.2. Overall Treatment Outcome Assessment (CGI-I)

The median CGI-I outcome one year after the start of intestinal levodopa infusion therapy was 2 (much improved), with a range between 2 (much improved) and 6 (much worse). Out of the 45 identified patients, 27 (60%) were much improved (CGI-I = 2) and were categorized as Responders. Another 15 patients (33%) were minimally improved (CGI-I = 3) and categorized as Partial Responders, and 3 patients (7%) were worse at one year (CGI-I > 3) and therefore categorized as Non-Responders.

### 3.3. Outcome Predictors

None of the 32 patients with OM OFF had a poor outcome: 10 had a partial response, and 22 were complete responders to LCIG treatment. Of the 13 patients without OM OFF, three had no improvement with LCIG, five were good responders and five were partial responders. The baseline variables for the different outcome groups: Responders (CGI-I = 2), Partial responders (CGI-I = 3) and Non responders (CGI-I > 3) are given in Table 2, together with the odds ratios and *p*-values calculated from ordinal regression. The factors and covariates were chosen based on the assumptions that infusion therapy would be more successful in patients with uncontrolled motor symptoms despite a high number of medication intakes and that sex and age may influence the outcome. The ordinal regression resulted in a significant fit (χ^2^ (10, 45) = 25.227, *p* = 0.005), indicating that at least one of the proposed predictors was related to the outcome. An increase in age by one year significantly reduced the odds for better outcome by 0.847, and the absence OM OFF periods in the PKG reduced the odds for good outcome by 0.055. No other baseline variables had a significant effect in the ordinal regression analysis.

### 3.4. Effect of LCIG Treatment

In the full population (*n* = 45), there was an overall increase in the median 24-h LED from 1233 mg at the time of the first PKG to 1520 mg at the outcome follow-up one year after treatment start (related samples Wilcoxon signed rank test, *p* = 0.004). The median daytime (06:00–22:00) levodopa-derived LED increased from 905 mg at the first PKG to 1356 mg after one year of treatment (related samples Wilcoxon signed rank test, *p* = 0.000). There was also an overall increase in the median LCIG dose from 1200 mg at the time of treatment initiation to 1356 mg after one year of treatment (related samples Wilcoxon signed rank test, *p* = 0.044).

A follow-up PKG recording was available for 39 of the patients at a median (min–max) of 4 (1–12) months after infusion treatment was started. In these patients, the summarized PKG measures BKS, DKS and FDS at follow-up were not significantly different from the baseline, but the change in FDS from 10.6 (4.5–25.8) to 8.6 (4.3–23.0) had a borderline *p*-value (Wilcoxon related samples signed rank, *p* = 0.056). Qualitative PKG assessments, however, improved in the responder group with both more absence of OM OFF periods and longer OM ON time. The total 24-h LED and daytime levodopa-derived LED also increased in the responder group at the time of follow-up. In the partial responder group, a significant increase was only observed for daytime levodopa-derived LED (Table 3).

## 4. Discussion

Almost all patients in this retrospective analysis of patients in West Sweden that had started treatment with LCIG between 2014 and 2020 were found to improve in the first year of LCIG treatment. However, a third of the patients had only a minimal improvement, and some worsened.

LCIG treatment is expensive and is associated with device-related complications [20,21], so information that can improve the identification of patients without or with less improvement would be valuable. The absence of clear identifiable OFF periods in the PKG recording and higher age were in the ordinal regression analysis associated with a less favorable clinical outcome after one year. Other results include a general increase in the treatment intensity and of the LCIG dose over the first treatment year. These changes were significant in the best responder group and may, in that group, reflect the amelioration of the OM OFF periods, as both the presence of OM OFF fluctuations and estimated OM ON time improved. The increase in treatment intensity was not associated with a significant change in DK scores in any of the outcome groups, and there was therefore no indication of increased hyperkinesia, in agreement with previous studies [2,22,23]. The nonresponding patients were too few for a reasonable statistical evaluation of the treatment intensity, but their 24-h LED at the baseline was approximately 30% higher than that of the partial and complete responders, and the LED did not increase with the infusion treatment. This could either indicate that the patients had already been optimized to an appropriate dose before the treatment or that the optimization after treatment was insufficient. However, the OM ON time was high already at the baseline in non-responders, so a ceiling effect is possible. If patients are carefully optimized with the best conventional treatment and can tolerate tablet intakes as frequently as every two hours, it is likely that improvement in the OFF time is limited. The beneficial effects of LCIG may, in such cases, be more related to reducing troublesome dyskinesia, improving the reliability of levodopa absorption, or simply removing the task of repeated medication intakes. It has unsurprisingly been shown also with the clinically assessed OFF time that patients with more OFF time at the baseline have more reduction in the OFF time with LCIG [24]. The same study from the GLORIA registry reported higher increases in LED in patients with more than 3 h OFF time at the baseline compared to patients with <3 h OFF, in line with what we observed, and found the OFF time at the baseline and disease duration to predict “robust” responders with a sustained reduction of OFF time of 3 h or more at 24 months after treatment start. The definition of a responder in that study differed from our definition, but our findings are coherent with their results.

All patients in the study were found to fulfil the criteria for advanced treatment after a team-based assessment and clinical conference with several movement disorder specialists experienced in all the available advanced treatments. The clinical routine since 2014 has been to include PKG recordings in this assessment, but the clinical interview has preference in the decision. This means that all patients in the study had convincingly described medication-related symptom fluctuations with a high impact on their quality of life. Despite this, not all of them displayed motor fluctuations that were detectable with the PKG device. As the PKG device is wrist-worn, some motor features cannot be expected to be detected, e.g., freezing of gait, axial dyskinesia and dystonia. Furthermore, non-motor symptoms other than sleep patterns can usually not be assessed. There is evidence that patients with motor fluctuations also improve regarding non-motor fluctuations after initiating LCIG treatment [5,25], but less is known when non-motor symptoms dominate, because such patients will, to a lesser extent, have been eligible for inclusion in LCIG RCTs where motor fluctuations are fundamental inclusion criteria.

There have been previous studies of objective measurement devices as tools for identifying patients that could benefit from device-assisted therapy. Heldman et al. reported a higher referral of patients to device-assisted therapy if the physician had access to objective monitoring in the home environment [26]. Data from the PKG has been used to design a classifier algorithm that effectively identifies patients that could benefit from device-assisted therapy or other changes in therapy [14]. This study differed in that the actual outcome of a device-assisted therapy was used instead of the earlier step of referral of patients for device-assisted therapy. We also used the PKG in a standard clinical fashion, with the ordinary summary scores combined with a qualitative assessment by a trained interpreter of PKG recordings. With this method, the assessment of OM ON time and OM OFF episodes is subjective, and this is an important limitation for interpreting the results. The interrater reliability of PKG ratings has not been studied much, but our experience is that the variability in qualitative ratings can be high. For example, when PKG recordings were assessed in a blinded fashion regarding the very basic question of whether two recordings were meaningfully different and which one represents the best situation, the agreement between two reviewers during the first review was only 67% [27]. Recently, a method for the automatic quantification of time with bradykinesia and time with hyperkinesia was suggested [28], and this can be valuable to use to verify if PKG measurements can predict the odds for success with device-assisted therapy. The correlation between diary-based assessments of motor states in PD and PKG measures is low to moderate [29], and the validity of PKG-based assessments of ON time and OFF time can therefore also be questioned. It is important to point out that OM OFF periods should not be used as a substitute for patient history or to challenge the patients experiences if there is a mismatch between the PKG and patient history or diaries. The findings that patients without OM OFF in the PKG had lower odds for success despite interview-verified motor fluctuations indicate that, even if the PKG measurements differ from the patients’ experiences, the objective data can hold information that predicts the outcome. Mapping clinical outcomes of interventions on the baseline objective monitoring is therefore expected to be more useful than improving the coherence between diaries and objective monitoring. It is, however, also clear that most patients without OM OFF periods in this study still experienced some improvement with LCIG, and not even the patients who were found to deteriorate after LCIG start terminated the treatment. When the outcome is less favorable than the patient’s expectation, it can be useful to advise the patient to return to conventional treatment for a week to determine if there is a difference.

The effect of age on odds for success was significant but small and is difficult to compare with the binary factor of OM OFF. The regression analysis indicates that the odds for good outcome decrease with 0.85 (0.73–0.98) per year. As the median age was 73 and the oldest pwPD was 85, the age effect would still be lower than the effect of no OM OFF (0.85^12^ = 0.14) for the oldest individual compared to the median. A recent matched case–control study compared the outcome of LCIG in pwPD 80 or older with pwPD younger than 75 and found no differences in the efficacy or safety of LCIG [30]. That study was designed to explicitly evaluate the effect of age on general and PD-specific outcome variables, and the matched control design will have eliminated confounding factors like comorbidity that were not accounted for in our study. A high chronologic age can therefore not be concluded to be an important predictor for a poorer LCIG outcome, despite the significant effect in our population.

There are some other limitations to this study. The sample size is small, and the explorative nature of the study increases the risk for type I errors. The outcome measure CGI-I is subjective, and the clinical and PKG assessments were made at varying time intervals in relation to the treatment start. It would have been valuable to support the CGI rating with UPDRS ratings and patient-reported quality of life. The availability of such data was, however, too patchy to allow an analysis. The CGI-I is usually used in a face-to-face meeting, and collateral information can be included. In this study, the assessment was based solely on collateral information, but this also facilitated unbiased comparisons with the baseline, which is sometimes difficult in a face-to-face assessment. At the time of the follow-up PKG, the LCIG treatment may not have been fully optimized. This could reduce the observed changes in the PKG measures but more so if PKGs were obtained to help analyze a perceived poor outcome. However, improvements seen with LCIG have been shown to start rapidly and to be stable over time [2]. Also, the PKG summary measures are not sensitive to variability of the motor states but more to the overall under/over treatment. The increase in PKG ON time and the reduced number of individuals with OM OFF fluctuations in the patient group with good outcomes verify that the clinical qualitative PKG assessment is also sensitive to change in the treatment effect regarding the variability of the motor states.

Many aspects are important in the selection of patients for device-assisted therapy and for the specific therapy modality. Cognitive function, non-motor complications like dysautonomia, social circumstances and personal preferences are aspects that must be considered. In the work-up for LCIG at our center, assessments include the Montreal Cognitive Assessment, MDS-UPDRS, wearing off questionnaires, the non-motor symptom questionnaire and anxiety/depression scales, as well as formal evaluations of treatment expectations from patients and caregivers. This information was, however, difficult to obtain from the medical records in many cases and was therefore not included in the evaluation of the predictive factors.

## 5. Conclusions

A retrospective analysis of 45 LCIG patients where PKG recordings had been obtained within a year before LCIG initiation indicated that increasing age and the absence of visually identifiable OFF periods in the PKG lowered the odds for treatment success. These findings should be confirmed with prospective studies with systematic and less subjective analyses of OM and of global outcomes. Some patients without OM OFF periods improved substantially, and the presence of OM OFF periods should therefore not be a condition for initiating LCIG. The absence of OM OFF periods can, however, be a reason for moderating patient and doctor expectations and to offer a trial period with a nasoduodenal tube before committing to surgical tube placement.

## Figures and Tables

**Table 1:** Baseline characteristics.

Baseline Variables	Median (Min–Max) or *n* (%)
Age (years)	73 (49–85)
LED_24h_ (mg)	1337 (677–3033)
Number of levodopa doses, 06:00–22:00	7 (5–9)
Levodopa dose interval (h)	2.5 (1.5–3)
Single dose effect duration (h) ^1^	2.0 (1.0–3.0)
OM ON-time, 06:00–22:00 ^1^	12 (4–16)
BK (median score 09:00–18:00)	22.8 (8.9–37.3.1)
DK (median score 09:00–18:00)	4.6 (0.6–46.3)
FDS	11.7 (4.5–25.8)
PTT (09:00–18:00), %	0.8 (0.1–46.5)
OM OFF episodes absent, *n* (%) ^1^	14 (31.1)

^1^ Determined by visual assessment of the PKG report by an experienced examiner (F.B.). Abbreviations: BK: bradykinesia score, DK: dyskinesia score, FDS: fluctuations dyskinesia score, PTT: percent time with tremor and OM: Objective Measure.

**Table 2:** Outcome predictive factors.

	Responders (CGI-I = 2) *n* = 27	Partial Responders (CGI-I = 3) *n* = 15	Non-Responders (CGI-I > 3) *n* = 3	Odds Ratio of a Good Outcome (95% Confidence Interval)	(Wald χ) *p*-Value
Age	70 (49–82)	78 (68–85)	73 (65–77)	0.847 (0.730–0.982)	(4.856) 0.028
Male, *n* (%)	19 (70.4)	8 (53.3)	2 (66.7)	6.194 (0.924–41.505)	(3.530) 0.060
Number of doses	7 (5–9)	6 (5–8)	7 (5–8)	1.010 (0.472–2.159)	(0.001) 0.980
BK	23.2 (8.9–37.3)	22.4 (13.4–30.4)	28.3 (16.6–36.3)	0.974 (0.783–1.212)	(0.057) 0.812
DK	4.5 (0.6–46.3)	4.6 (1.1–17.9)	5.5 (0.8–11.4)	0.976 (0.764–1.246)	(0.039) 0.844
FDS	11.7 (4.5–25.8)	12.2 (7.9–20.6)	9.3 (5.7−10.6)	1.124 (0.699–1.806)	(0.232) 0.630
PTT (%)	0.9 (0.1–46.5)	0.5 (0.1–0.3)	0.6 (0.1–0.9)	1.255 (0.692–2.276)	(0.559) 0.455
OM ON-time	11 (8–16)	12 (4−16)	12 (12–16)	1.449 (0.898–2.339)	(2.309) 0.129
No OM OFF, *n* (%)	5 (18.5)	5 (31.1)	3 (100)	0.055 (0.003–0.884)	(4.188) 0.041
LED_24h_, mg	1235 (677–2267)	1184 (712–3034)	1514 (1308–2300)	0.240 ^1^ (0.018–3.278)	(1.144) 0.285

^1^ The natural logarithms of the LED values were used in the regression model, but absolute values are given for the three outcome groups in the table.

**Table 3:** Outcome measures in the three clinical outcome groups.

Outcome Group		BK	DK	FDS	PTT (%)	OM ON Time	No OM OFF, *n*	LED_24h_	LED_16h_ ^2^
Responders	Baseline	24.1	4.3	10.3	1.3	11	5	1235	850
		(8.9–37.3)	(0.6–46.3)	(4.5–25.8)	(0.1–46.5)	(8–16)		(677–2267)	(499–1697)
	Follow-up	23.6	4.1	9.6	0.9	14	18	1479	1376
		(10.2–35.5)	(0.9–36.5)	(5.7–23.0)	(0.0–13.8)	(4–16)		767−2374	(330–1964)
	*p*-value ^1^ *(n)*	0.412	0.893	0.333	0.213	0.045	0.000	0.005	0.000
		(25)	(25)	(25)	(25)	(25)	(25)	(27)	(27)
Part. responders	Baseline	18.4	9.8	14	0.5	13	5	1184	900
		(13.4–30.8)	(1.6–17.9)	(7.9–20.6)	(1.0–3.0)	(6–16)		(712–3034)	(450–1663)
	Follow-up	21.8	6.4	9.1	0.4	13	6	1382	1040
		(10.3–33.9)	(0.6–17.6)	(5.7–19.8)	(0.2–10)	(6–16)		(588–2195)	(528–1764)
	*p*-value ^1^ *(n)*	0.093	0.197	0.155	0.266	0.345	1.000	0.156	0.009
		(11)	(11)	(11)	(11)	(11)	(11)	(15)	(15)
Non-responders	Baseline	28.3	5.5	9.3	0.6	12	3	1514	1614
		(16.6–36.3)	(0.8–11.4)	(5.7–10.6)	(0.1–0.9)	(12–16)		(1308–2300)	(1540–1620)
	Follow-up	35.6	0.6	5.7	0.7	7	3	1364	1420
		(26.5–42.1)	(0.2–0.9)	(4.3–8.10)	(0.2–2.4)	(2–15.5)		(998–1600)	(1156–1454)

^1^ Nonparametric tests: McNemar-related samples or Wilcoxon-related samples signed rank test. ^2^ Oral medication including levodopa and enzyme inhibitors at the baseline and intestinal infusion dose at follow-up. Abbreviations: Levodopa: LD, Levodopa equivalent dose: LED, Bradykinesia score: BKS, Dyskinesia score: DKS, Fluctuation and Dyskinesia Score: FDS, OM OFF: PKG based measurement of existence of apparent of motor OFF fluctuations. OM ON time: PKG based visual assessment of total time without OFF periods between 06:00 and 22:00. Only subjects where follow-up measures were available are included. The No responder group was too small for statistical tests.

## Data Availability

The data presented in this study are available on request from the corresponding author. The data are not publicly available due to the risk of breached patient confidentiality.
